# Multiple Serum Cytokine Profiling to Identify Combinational Diagnostic Biomarkers in Attacks of Familial Mediterranean Fever

**DOI:** 10.1097/MD.0000000000003449

**Published:** 2016-04-22

**Authors:** Tomohiro Koga, Kiyoshi Migita, Shuntaro Sato, Masataka Umeda, Fumiaki Nonaka, Shin-Ya Kawashiri, Naoki Iwamoto, Kunihiro Ichinose, Mami Tamai, Hideki Nakamura, Tomoki Origuchi, Yukitaka Ueki, Junya Masumoto, Kazunaga Agematsu, Akihiro Yachie, Koh-Ichiro Yoshiura, Katsumi Eguchi, Atsushi Kawakami

**Affiliations:** From the Unit of Translational Medicine, Department of Immunology and Rheumatology, Nagasaki University Graduate School of Biomedical Sciences (TK, MU, FN, S-YK, NI, KI, MT, HN, TO, AK), Nagasaki; Department of Rheumatology and Clinical Research Center (KM), Nagasaki Medical Center, Omura; Nagasaki University Hospital (SS), Clinical Research Center, Nagasaki; Department of Internal Medicine (FN), Sasebo City General Hospital; Center for Rheumatic Disease (YU, KE), Sasebo Chuo Hospital, Sasebo; Department of Pathology (JM), Ehime University Graduate School of Medicine and Proteo-Science Center, Toon, Ehime; Department of Infectious Immunology (KA), Shinshu University, Graduate School of Medicine, Matsumoto; Department of Pediatrics (AY), School of Medicine, Institute of Medical, Pharmaceutical and Health Sciences, Kanazawa University, Kanazawa; and Department of Human Genetics (K-IY), Nagasaki University Graduate School of Biomedical Sciences, Nagasaki, Japan.

## Abstract

The precise cytokine networks in the serum of individuals with familial Mediterranean fever (FMF) that are associated with its pathogenesis have been unknown. Here, we attempted to identify specific biomarkers to diagnose or assess disease activity in FMF patients.

We measured serum levels of 45 cytokines in 75 FMF patients and 40 age-matched controls by multisuspension cytokine array. FMF in “attack” or “remission” was classified by Japan College of Rheumatology-certified rheumatologists according to the Tel Hashomer criteria. Cytokines were ranked by their importance by a multivariate classification algorithm. We performed a logistic regression analysis to determine specific biomarkers for discriminating FMF patients in attack. To identify specific molecular networks, we performed a cluster analysis of each cytokine.

Twenty-nine of the 45 cytokines were available for further analyses. Eight cytokines’ serum levels were significantly elevated in the FMF attack versus healthy control group. Nine cytokines were increased in FMF attack compared to FMF remission. Multivariate classification algorithms followed by a logistic regression analysis revealed that the combined measurement of IL-6, IL-18, and IL-17 distinguished FMF patients in attack from the controls with the highest accuracy (sensitivity 89.2%, specificity 100%, and accuracy 95.5%). Among the FMF patients, the combined measurement of IL-6, G-CSF, IL-10, and IL-12p40 discriminated febrile attack periods from remission periods with the highest accuracy (sensitivity 75.0%, specificity 87.9%, and accuracy 84.0%).

Our data identified combinational diagnostic biomarkers in FMF patients based on the measurement of multiple cytokines. These findings help to improve the diagnostic performance of FMF in daily practice and extend our understanding of the activation of the inflammasome leading to enhanced cytokine networks.

## INTRODUCTION

Familial Mediterranean fever (FMF) is an inherited autoinflammatory disorder characterized by unpredictable attacks of fever with arthritis, abdominal pain, and/or serositis.^1,2^ The usual clinical manifestations of FMF are acute episodes of inflammation and there is no residual symptom between attacks. However, chronic subclinical inflammation may persist despite achieving remission.^3^ FMF is associated with a number of mutations of the *Mediterranean fever (MEFV)* gene, which codes for a protein named pyrin. Pyrin acts as a major regulatory component of the NACHT, LRR, and PYD domains-containing protein 3 inflammasome complex.^4^ Dysfunction of pyrin causes autoinflammatory disease, resulting in the aberrant production of interleukin (IL)-1β and IL-18.^5,6^ These cytokines activate nuclear factor κB signaling pathways that lead to increased amounts of tumor necrosis factor-alpha (TNF-α) and IL-6.^7,8^

In line with these observations, it is widely known that elevated acute-phase proteins such as serum amyloid A and C-reactive protein and inflammatory cytokines including IL-6 and IL-18 are implicated in the disease activity of FMF in clinical practice.^3,9,10^ However, a specific biomarker for FMF is not yet available, and the cytokine profile in serum from FMF patients in attacks associated with its pathogenesis has not been established. There has been no extensive study examining multiple cytokines and investigating their importance and pathogenic networks.

In the present study, in order to identify the utility of the measurement of multiple cytokines including a specific combination of biomarkers for clinical application, we analyzed the serum from FMF patients in attack or in remission by using a multisuspension cytokine array. Our findings demonstrated that the serum from FMF patients in attack had higher levels of several inflammatory cytokines compared to the serum from FMF patients in remission and a normal population. We also identified a specific combination of cytokines that distinguished FMF patients in attack from those in remission and the normal population.

## METHODS

### Patients and Controls

This study was registered with the University Hospital Medical Information Network Clinical Trials Registry (http://www.umin.ac.jp/ctr/) as UMIN000015881. The study population consisted of 75 Japanese patients with FMF who were recruited consecutively and prospectively between May 2010 and October 2015 from Nagasaki University, Shinshu University, Kanazawa University and Nagasaki Medical Center. Each of the FMF patients fulfilled the Tel Hashomer criteria.^11,12^ All participants undergo a clinical assessment and provide a blood sample for the assay at the same time. The control group was 40 age- and sex-matched healthy Japanese individuals recruited from staff at Nagasaki University. Sixty-four of the 75 patients (84%) expressed a typical FMF attack, and the remaining 11 patients (16%) expressed an incomplete attack as defined by the Tel Hashomer criteria.^11^

The distribution of the *MEFV* genotype was as follows: M694I/M694I, 5 patients; M694I/–, 7; M694V/–, 3; M694I/E148Q, 23; M694I/P715L, 1; M694I/E148Q/L110P, 7; M680I/V726A, 1; E148Q/–, 6; L110P/E148Q/E148Q, 1; L110P/E148Q, 4; L110P/E148Q/R202Q, 2; L110P/E148Q/P369S, 1; E148Q/P369S/R408Q, 2; E84K/–, 2, E84K/G304R, 1; E84K/R410H, 1; G304R/–, 1; S503C/–, 1; no mutation, 6 patients.

Serum samples were centrifuged at 3000 *g* for 5 minutes, and the supernatants were collected and stored at –80 °C before the assay was performed. We obtained 82 serum samples from FMF patients in total (21 patients for attack only, 47 patients for remission only, and 7 patients for both attack and remission; thus, 28 samples in attack and 54 samples in remission). All patients gave their signed informed consent to be subjected to the protocol, which was approved by the Institutional Review Board of Nagasaki University and related centers (Approval No. 14092946).

### Multiplex Cytokine Assay

We performed a multiplex cytokine bead assay blindly and in parallel using the Bio-plex Pro Human Cytokine assay (Bio-Rad, Hercules, CA) and Milliplex MAP Human Cytokine/Chemokine panel 1 (Millipore, Billerica, MA) according to the manufacturers’ instructions. We were able to further analyze 29 of the 45 cytokines: IL-1β, IL-1 receptor antagonist (IL-1RA), IL-2, IL-4, IL-5, IL-6, IL-7, IL-8, IL-10, IL-12 (p40), IL-12 (p70), IL-17, IL-18, TNF-α, interferon-α (IFN-α), IFN-γ, granulocyte macrophage colony stimulating factor, basic granulocyte colony stimulating factor (G-CSF), vascular endothelial growth factor, soluble CD54 (sCD54), sCD106, fibroblast growth factor 2, CCL2 (monocyte chemoattractant protein-1/MCAF), CCL3 (macrophage inflammatory protein-1a), CCL4 (macrophage inflammatory protein-1b), CCL22 (human macrophage-derived chemokine), CXCL1 (growth-regulated protein alpha precursor), CXCL10 (IFN-γ inducible protein 10), and CX3CL1 (fractalkine).

### Statistical Analysis

The subjects’ baseline demographic characteristics were compared using Fisher exact tests for discrete variables and Wilcoxon test for continuous variables. The Kruskal–Wallis test followed by a Dunn multiple comparisons test was used to compare cytokine levels between groups. To rank the cytokine levels, we performed a multivariate classification algorithm termed random forest analysis (RFA),^13^ using the R package RandomForest (http://cran.r-project.org/web/packages/randomForest/) ver. 4.6–12 software, as described.^14^ We subsequently selected a classifier consisting of a combination of cytokine markers yielding the best classification performance to predict FMF attacks by a multiple logistic regression analysis. We then calculated the sensitivity, specificity, accuracy, a receiver operator characteristic curve, the area under the curve, and Akaike information criterion. Correlations between pairs of serum markers were calculated using Spearman rank correlation test. We used Wilcoxon signed-rank tests to evaluate the continuous change of cytokines between FMF in attack and FMF in remission. The cytokine profiles of the FMF patients with or without mutations in exon 10 were compared with Wilcoxon test. Statistical analyses were performed in R (ver. 3.2.3) and JMP pro 11.2 software (SAS Institute, Cary, NC). All reported *P*-values are 2-sided. A *P*-value <0.05 was considered significant.

## RESULTS

### Cytokine Profiles of the FMF Patients and the Healthy Controls

Table 1 shows the characteristics of the FMF patients. Out of the 75 patients, 48 patients (64%) were female. The patients’ median age at onset was 22 years, and their median age at diagnosis was 38 years. We found that the serum levels of 8 cytokines (IL-4, IL-6, IL-7, IL-17, IL-18, G-CSF, sCD54, and CXCL10) were significantly elevated in the FMF attack group compared to the healthy control group. In addition, 9 cytokines (IL-6, IL-8, IL-10, IL-12p40, IFN-α, IFN-γ, G-CSF, sCD106, and CXCL10) were significantly increased in the FMF attack group compared to the FMF patients in remission (Table 2).

**TABLE 1:**
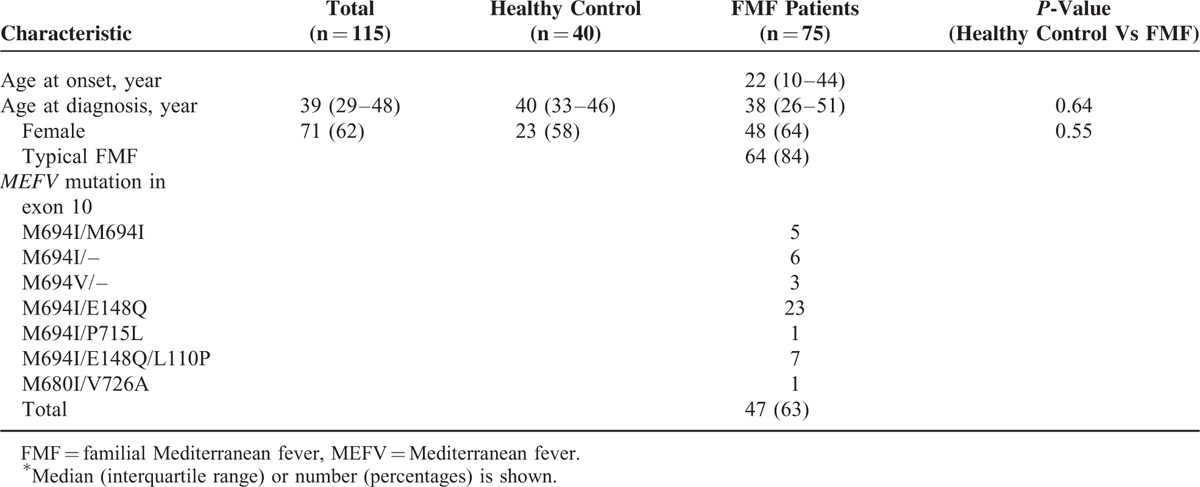
Patients Demographic Profile^∗^

**TABLE 2:**
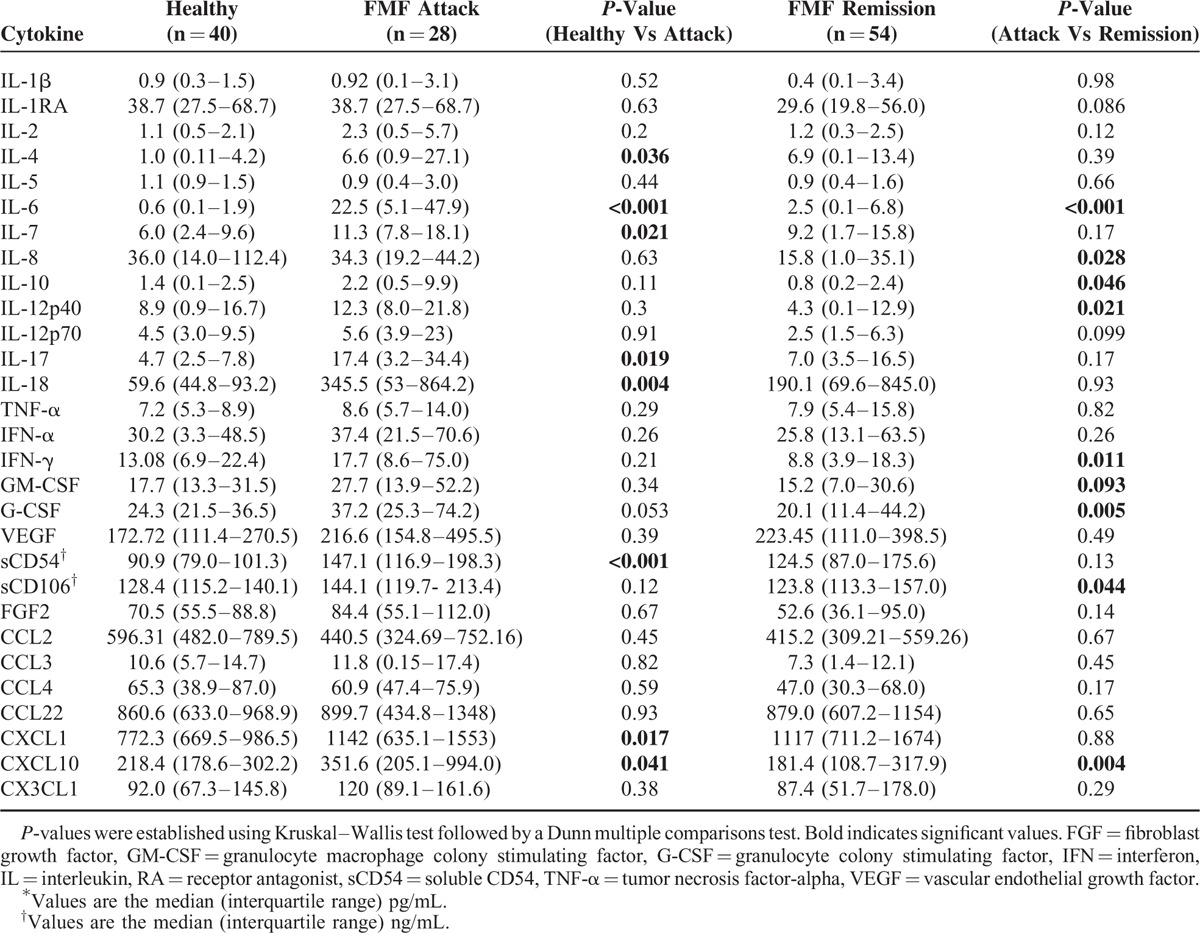
Cytokine Profile of FMF Patients and Healthy Control^∗^

### Identification of Combinational Biomarkers Specific for FMF in Attack by the Random Forest Analysis and Logistic Regression Analysis

**FIGURE 1:**
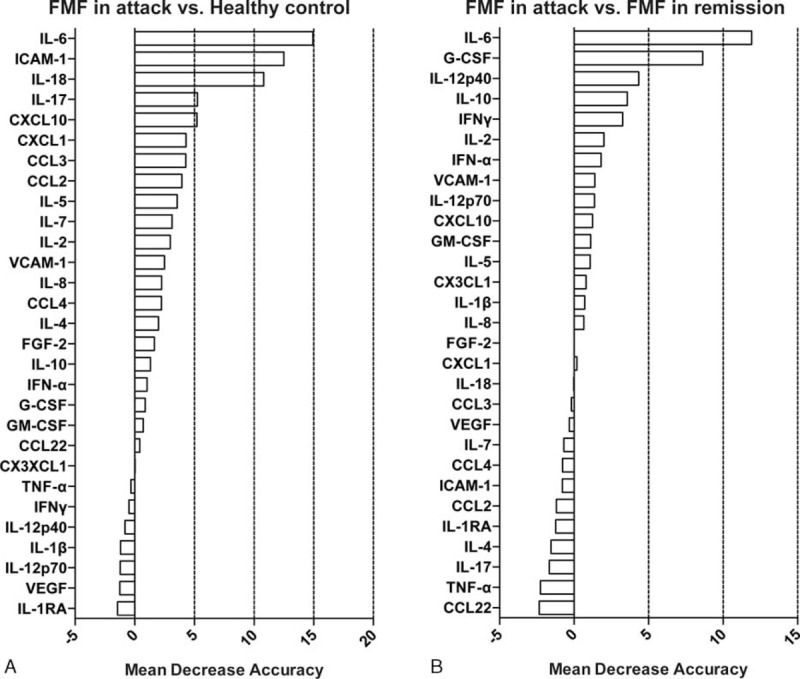
RFA, cytokines are ranked by their relative importance for discriminating FMF in attack from healthy subjects (A) or FMF in remission (B). The horizontal axis represents the average decrease in classification accuracy. FMF = familial Mediterranean fever, RFA = random forest analysis.

**FIGURE 2:**
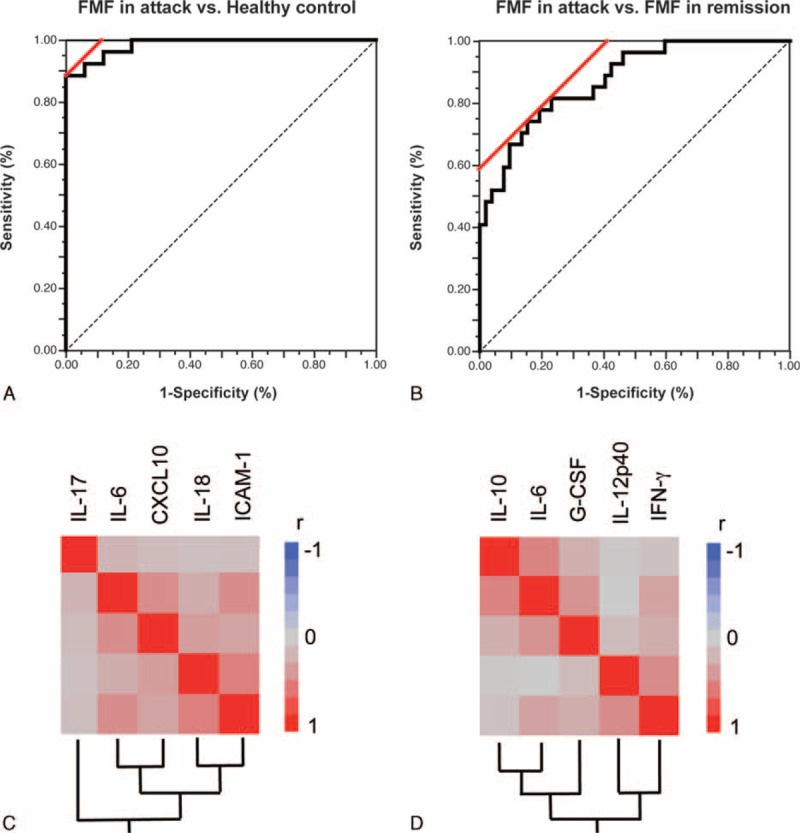
ROC curve analysis for the prediction of FMF in attack by a specific set of cytokines. (A) Healthy control versus FMF in attack; the combined measurement of IL-6, IL-18, and IL-17. (B) FMF in remission versus FMF in attack; the combined measurement of IL-6, G-CSF, IL-12p40, and IL-10. Hierarchical clustering with a Spearman correlation heat map of serum cytokine levels among (C) the FMF in attack and healthy control groups and (D) the FMF in attack and FMF in remission groups. FMF = familial Mediterranean fever, G-CSF = granulocyte colony stimulating factor, IL = interleukin, ROC receiver operator characteristic.

To determine the most important predictor that can discriminate FMF in attack from FMF in remission or healthy controls, we ranked the cytokines by their importance, using the RFA (Figure 1A, healthy controls vs FMF in attack; Figure 1B, FMF in remission vs FMF in attack)(see Supplementary Digital Content). We subsequently performed a multiple logistic regression analysis and constructed receiver operator characteristic curves to calculate the sensitivity, specificity, accuracy, area under the curve, and Akaike information criterion. Table 3 shows these variables in each combination. The data revealed that IL-6, IL-18, and IL-17 were the best combination to distinguish FMF patients in attack from the normal population with high accuracy (sensitivity 89.2%, specificity 100%, and accuracy 95.5%, Figure 2A). The prediction of attack used the following formula:

**TABLE 3:**
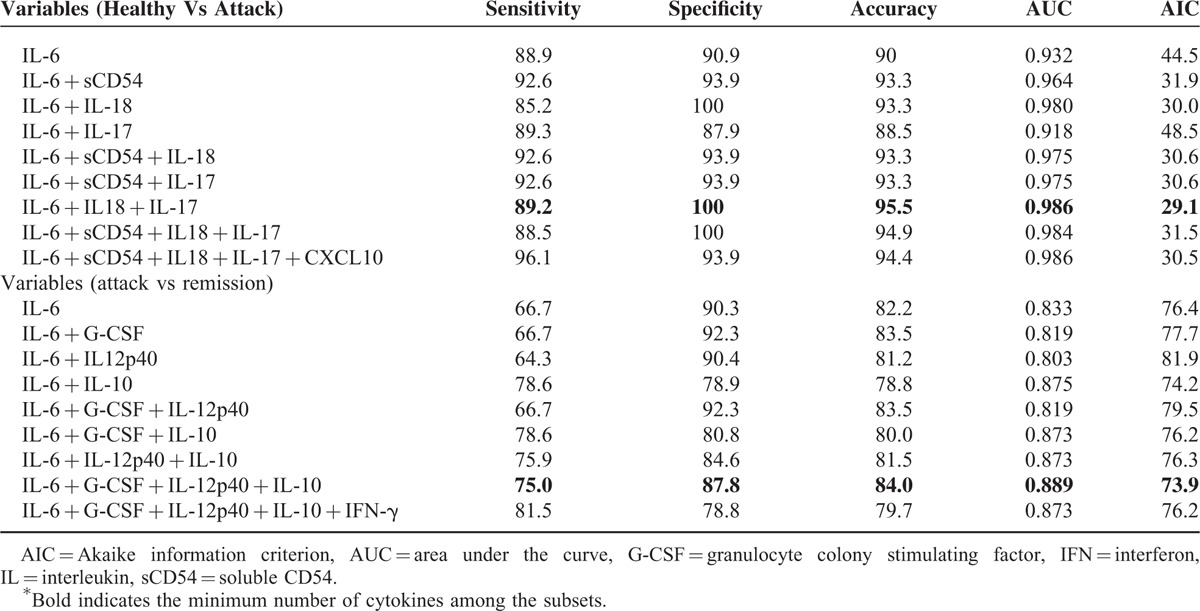
ROC Curve in Each Subset Determined by Multiple Logistic Regression Analysis^∗^

Probability (attack) = exp(A)/{+exp(A)} [A = 4.208 − 0.318^∗^IL-6 − 0.010^∗^IL-18 − 0.037^∗^IL-17].

As shown in Figure 2B, we also observed that the combined measurement of IL-6, G-CSF, IL-12p40, and IL-10 discriminated the attack periods from the remission periods with the highest accuracy (sensitivity 75.0%, specificity 87.9%, and accuracy 84.0%) The prediction of attack used the following formula:

Probability (attack) = exp(A)/{1 + exp(A)} [A = 2.550 − 0.085^∗^IL-6 − 0.002^∗^G-CSF − 0.001^∗^IL-12p40 − 0.231^∗^IL-10].

### Activated Cytokine Networks Among the FMF in Attack and Healthy Control Groups and Among the FMF in Attack and FMF in Remission Groups

To identify activated cytokine networks of FMF in attack, we further examined the correlations between the serum concentrations of the top 5 individual cytokines ranked by the RFA. In the FMF in attack and healthy control groups, there were significant correlations between sCD54 and IL-18 (*r* = 0.547, *P* = 0.010), IL-6 and sCD54 (*r* = 0.487, *P* < 0.001), CXCL10 and sCD54 (*r* = 0.343, *P* = 0.006), CXCL10 and IL-6 (*r* = 0.474, *P* = 0.001), and CXCL10 and IL-18 (*r* = 0.559, *P* = 0.005).

In the FMF in attack and FMF in remission groups, there were significant correlations between G-CSF and IL-6 (*r* = 0.463, *P* < 0.001), IL-6 and IL-12p40 (*r* = 0.282, *P* = 0.040), IL-10 and IL-6 (*r* = 0.531, *P* < 0.001), IL-10 and IL-12p40 (*r* = 0.731, *P* < 0.001), IL-10 and G-CSF (*r* = 0.494, *P* = 0.047), IFN-γ and IL-12p40 (*r* = 0.457, *P* < 0.001), IFN-γ and G-CSF (*r* = 0.276, *P* = 0.012), and IFN-γ and IL-6 (*r* = 0.356, *P* = 0.0010). Hierarchical clustering with heatmaps based on the Spearman correlation coefficients are shown in Figure 2C (healthy control and attack) and D (attack and remission).

### Validation Analysis of the Combinational Biomarkers in the Serial Serum Samples From the FMF Patients

**FIGURE 3:**
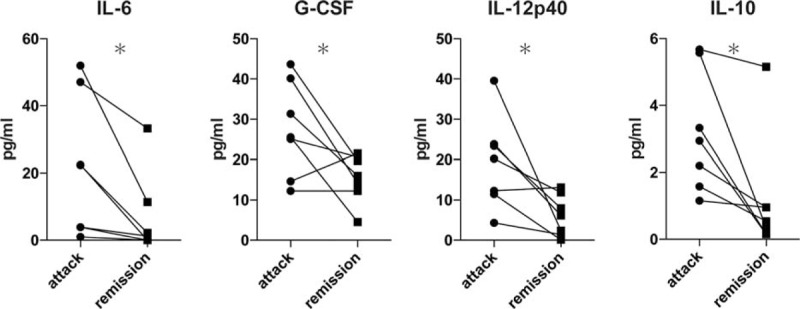
Serial cytokine changes in attack and in remission. The lines link the same patients. The changes from baseline were compared using Wilcoxon signed rank test (^∗^*P* < 0.05).

To determine whether the identified combinational biomarkers in this study are also relevant in serial serum samples, we analyzed serial cytokine changes in attack and in remission in 7 patients with FMF. As shown in Figure 3, the serum levels of IL-6, G-CSF, IL-12p40, and IL-10 were significantly decreased in FMF remission compared to FMF attack.

### Comparison of *MEFV* Genotypes With Cytokine Profiling

Since it was proposed that mutations at position 680 to 694 in exon 10 present a risk of severe FMF,^15^ we investigated whether these mutations can affect the levels of cytokines. To this end, we compared 7 important cytokines of FMF in attack and in remission determined by the RFA in the absence or presence of *MEFV* mutation in exon 10. As shown in Table 4, no significant difference was observed among these cytokines in attack. In contrast, the serum level of IL-18 in remission was significantly higher in the patients with *MEFV* mutation in exon 10 (Table 4).

**TABLE 4:**
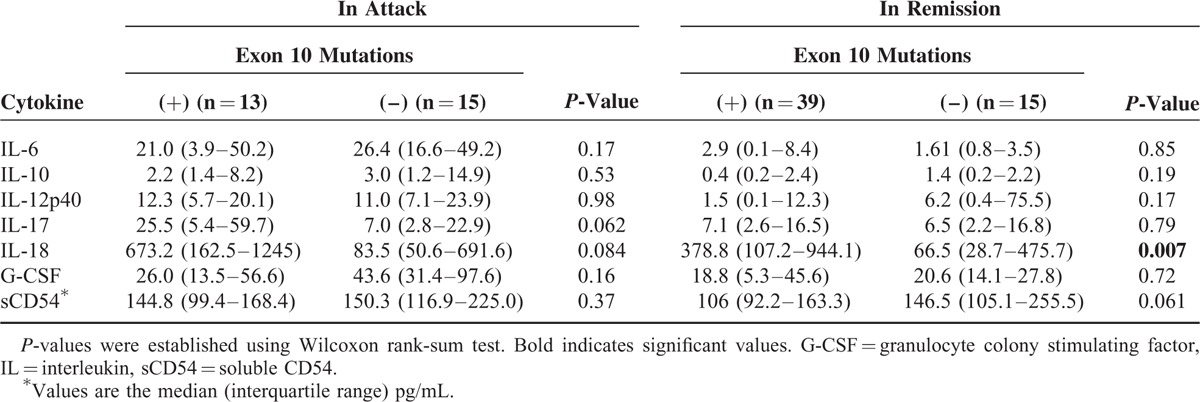
Comparison of Cytokine Profile in FMF With or Without Mutations in Exon 10^∗^

## DISCUSSION

FMF is a hereditary autoinflammatory disease caused by the mutation of pyrin, which is involved in inflammasome complex formation.^16^ Accordingly, activated cytokine networks are implicated in the pathogenesis of FMF.^3,10,17^ However, these prior studies focused on a few individual cytokines in FMF. Our present work suggested cytokine networks in FMF patients by using a multisuspension cytokine array system, and we were able to identify a set of possible diagnostic and disease activity markers for FMF with high accuracy.

IL-6 is an inflammatory cytokine that plays a pivotal role in autoimmune and chronic inflammatory diseases. Increased levels of IL-6 in the serum during FMF attacks were reported.^10,17,18^ Our present findings demonstrated that IL-6 had the best performance for distinguishing FMF in attack from healthy controls or FMF in remission. Other recent case reports have shown the efficacy of an IL-6 inhibitor in clinical practice for colchicine-resistant FMF or secondary amyloidosis in FMF patients.^19–22^ Taken together, our data support the notion of IL-6 as a main inflammatory cytokine in FMF and, thus, as a promising target in this disease.

Inflammasomes can activate caspase-1, which processes pro-IL-1β and pro-IL-18 from their inactive to mature and biologically active forms^23^ and these cytokines are closely associated with T-helper (Th) cell differentiation.^24^ The abnormal regulation of the innate immune response and Th17 cell polarization via IL-1 signaling plays pathogenic roles in the development of autoinflammatory diseases including FMF.^25^ On the other hand, IL-18 has the ability to induce Th1 cell differentiation by enhancing INF-γ production.^26,27^ As also observed in an earlier study,^28^ our findings demonstrated that the serum IL-17 and IL-18 levels of FMF patients both in attack and in remission were significantly higher than those of healthy controls, and that the levels of serum IL-17 and IL-18 were comparable in FMF patients in attack and in remission.

In addition, CXCL10, IL-12p40, and IFN-γ, which are known as Th1-related chemokines and cytokines,^29–31^ were significantly elevated in FMF patients in attack. The data obtained in the present study indicate that aberrant Th1/Th17 differentiation or activation is involved in acute and subclinical chronic inflammation in FMF. A plausible explanation is thus that the combination of IL-17 and IL-18 with IL-6 is very characteristic in activated cytokine patterns for FMF patients in attack.

We observed that the serum levels of sCD54 were significantly higher in the FMF attack group compared to the healthy controls but not significantly higher than the levels of the FMF patients in remission. This finding suggests that CD54 is involved in the development of FMF and that it could be useful as a diagnostic marker. CD54 is an intercellular adhesion molecule expressed mainly by the vascular endothelium, macrophages, and lymphocytes. This protein can be induced by pathogenic cytokines related to autoinflammatory diseases, such as IL-1β, IL-18, and TNF-α.^32–34^ Indeed, it has been reported that the level of sCD54 was increased in the serum from patients with FMF and patients with Adult Still Disease.^35,36^ These finding are consistent with our present observation that sCD54 clearly correlates with IL-18 in the serum from FMF patients in attack.

In our comparison of the FMF patients in attack and those in remission, we found highly elevated serum levels of IL-6, G-CSF, IL-12p40, and IL-10. Although elevated G-CSF in the serum from FMF patients had not been reported previously, Stojanov et al observed that the serum level of G-CSF was significantly higher in patients with periodic fever, aphthous stomatitis, pharyngitis, and adenitis (PFAPA) attacks.^37^ We also detected the elevated expression of CD64 on neutrophils in patients with FMF.^38,39^ These observations support the importance of G-CSF in FMF attack.

IL-10, considered a regulator of inflammation, was also elevated in FMF attack. Elevated serum levels of IL-10 along with proinflammatory cytokines were reported in FMF patients in attack^10^ and other inflammatory diseases or macrophage activation syndrome.^40–43^ Accordingly, this increase in IL-10 could be partly explained by a counter response that functions to regulate the aberrant production of inflammatory cytokines.

IL-12, a heterodimeric protein of two subunits (p35, p40; p70 is a heterodimer of p35 and p40) secreted by phagocytic cells in response to pathogens, is crucial in Th1 cell polarization.^44^ Increased levels of IL-12 in the serum of FMF patients in both attack and remission were reported,^3,45^ but the distinction of each subunit was not clear in the previous reports, and our present data showed the difference in the IL-12p40 subunit only. The increase in serum IL-12 is considerable among FMF patients in attack. In addition, although only 7 patients were available for our examination of the cytokine profiles in both attack and remission, all 4 of the above-mentioned cytokines (IL-6, G-CSF, IL-12p40, and IL-10) in remission are decreased in total or individually. Accordingly, we speculate that the serum levels of these combinational biomarkers would be appreciable in cases of serial measurements in FMF patients.

Although the efficacy of an IL-1 inhibitor and a TNF inhibitor in severe FMF patients has been shown,^46^ we could not find any significant differences in these cytokines among our patients with FMF. Possible reasons for this discrepancy are that: the amount of the detectable soluble form of IL-1β is limited due to the intracellular localization of pro-IL-1β or binding to target proteins such as IL-1RA in the serum; IL-1β and TNF-α are produced mainly in inflamed local tissues; and the concentration of these cytokines in the serum is not great enough for the assay that we used to detect significant differences. Thus, we cannot exclude the possibility that the contributions of IL-1β and TNF-α to the pathogenesis of FMF are underestimated in the present study.

We also attempted to determine whether cytokine levels are affected by *MEFV* exon 10 mutation, as an association between *MEFV* exon 10 mutation and subclinical inflammation in FMF patients was described.^3^ In the present study, we found that there was no significant difference in 7 important cytokines in FMF attack. These observations indicate that the mutation in exon 10 does not affect cytokine levels in attack among Japanese FMF patients and that the utility of combinational biomarkers is assured regardless of *MEFV* gene mutations.

Importantly, our findings also showed that serum IL-18 in remission was significantly higher in the FMF patients with *MEFV* exon 10 mutation than in those without mutation, whereas other cytokines were not significantly different in either attack or remission. A 2014 study of Japanese FMF patients also demonstrated that the serum IL-18 level in FMF patients in remission was significantly higher in typical FMF with M694I mutation than in atypical FMF with exon2 variants including E148Q.^47^ Based on our present data and previous findings, we speculate that in FMF, a pathogenic mutation in exon 10 facilitates IL-18-dependent subclinical inflammation via an aberrant regulation of the pyrin inflammasome cascade.

Our study has several limitations. Serial serum sampling was possible for only some of the patients. Therefore, large validation studies are required to characterize the performance of this assay. In addition, a considerable number of our patients in remission were treated with colchicine, the mode of action of which is to inhibit the NALP3 inflammasome.^48^ Thus, inflammatory cytokines in remission could be pharmacologically suppressed by colchicine, and this may not reflect the nature of cytokine networks of FMF patients in remission. However, no large-scale, comprehensive analysis has been conducted to test the findings of past studies and clarify the association between each pathogenic cytokine in FMF. Our present data show, for the 1st time, specific cytokine networks in FMF. These findings help to improve the diagnostic performance of FMF in daily practice and facilitate our understanding of the inflammatory mechanisms of FMF patients.

## Supplementary Material

Supplemental Digital Content

## References

[1] Federici S Sormani MP Ozen S Evidence-based provisional clinical classification criteria for autoinflammatory periodic fevers. *Ann Rheum Dis* 2015; 74:799–805. 25637003 10.1136/annrheumdis-2014-206580

[2] Ben-Chetrit E Levy M Familial Mediterranean fever. *Lancet* 1998; 351:659–664. 9500348 10.1016/S0140-6736(97)09408-7

[3] Ben-Zvi I Livneh A Chronic inflammation in FMF: markers, risk factors, outcomes and therapy. *Nat Rev Rheumatol* 2011; 7:105–112. 21060333 10.1038/nrrheum.2010.181

[4] Stehlik C Reed JC The PYRIN connection: novel players in innate immunity and inflammation. *J Exp Med* 2004; 200:551–558. 15353551 10.1084/jem.20032234 PMC2212741

[5] Kim ML Chae JJ Park YH Aberrant actin depolymerization triggers the pyrin inflammasome and autoinflammatory disease that is dependent on IL-18, not IL-1beta. *J Exp Med* 2015; 212:927–938. 26008898 10.1084/jem.20142384 PMC4451132

[6] Chae JJ Cho YH Lee GS Gain-of-function Pyrin mutations induce NLRP3 protein-independent interleukin-1beta activation and severe autoinflammation in mice. *Immunity* 2011; 34:755–768. 21600797 10.1016/j.immuni.2011.02.020 PMC3129608

[7] Hoffman HM Therapy of autoinflammatory syndromes. *J Allergy Clin Immunol* 2009; 124:1129–1138.quiz 1139–1140. 20004774 10.1016/j.jaci.2009.11.001 PMC4508191

[8] Bagci S Toy B Tuzun A Continuity of cytokine activation in patients with familial Mediterranean fever. *Clin Rheumatol* 2004; 23:333–337. 15293095 10.1007/s10067-004-0925-4

[9] Oktem S Yavuzsen TU Sengul B Levels of interleukin-6 (IL-6) and its soluble receptor (sIL-6R) in familial Mediterranean fever (FMF) patients and their first degree relatives. *Clin Exp Rheumatol* 2004; 22 (4 Suppl 34):S34–36. 15515781

[10] Manukyan GP Ghazaryan KA Ktsoyan Zh A Cytokine profile of Armenian patients with Familial Mediterranean fever. *Clin Biochem* 2008; 41:920–922. 18440310 10.1016/j.clinbiochem.2008.03.017

[11] Livneh A Langevitz P Zemer D Criteria for the diagnosis of familial Mediterranean fever. *Arthritis Rheum* 1997; 40:1879–1885. 9336425 10.1002/art.1780401023

[12] Berkun Y Eisenstein EM Diagnostic criteria of familial Mediterranean fever. *Autoimmun Rev* 2014; 13:388–390. 24424166 10.1016/j.autrev.2014.01.045

[13] Breiman L Random forests. *Mach Learn* 2001; 45:5–32.

[14] Kokkonen H Soderstrom I Rocklov J Up-regulation of cytokines and chemokines predates the onset of rheumatoid arthritis. *Arthritis Rheum* 2010; 62:383–391. 20112361 10.1002/art.27186

[15] Giancane G Ter Haar NM Wulffraat N Evidence-based recommendations for genetic diagnosis of familial Mediterranean fever. *Ann Rheum Dis* 2015; 74:635–641. 25628446 10.1136/annrheumdis-2014-206844

[16] Savic S Dickie LJ Battellino M Familial Mediterranean fever and related periodic fever syndromes/autoinflammatory diseases. *Curr Opin Rheumatol* 2012; 24:103–112. 22089100 10.1097/BOR.0b013e32834dd2d5

[17] Akcan Y Bayraktar Y Arslan S The importance of serial measurements of cytokine levels for the evaluation of their role in pathogenesis in familial Mediterraean fever. *Eur J Med Res* 2003; 8:304–306. 12911867

[18] Gang N Drenth JP Langevitz P Activation of the cytokine network in familial Mediterranean fever. *J Rheumatol* 1999; 26:890–897. 10229412

[19] Umeda M Aramaki T Fujikawa K Tocilizumab is effective in a familial Mediterranean fever patient complicated with histologically proven recurrent fasciitis and myositis. *Int J Rheum Dis* 2015; DOI: 10.1111/1756-185X.12776. 10.1111/1756-185X.12776 26481326

[20] Fujikawa K Migita K Tsukada T Interleukin-6 targeting therapy in familial Mediterranean fever. *Clin Exp Rheumatol* 2013; 31 (3 Suppl 77):150–151. 24064027

[21] Hamanoue S Suwabe T Hoshino J Successful treatment with humanized anti-interleukin-6 receptor antibody (tocilizumab) in a case of AA amyloidosis complicated by familial Mediterranean fever. *Mod Rheumatol* 2015; 1–4. 25619282 10.3109/14397595.2014.908810

[22] Yilmaz S Cinar M Simsek I Tocilizumab in the treatment of patients with AA amyloidosis secondary to familial Mediterranean fever. *Rheumatology (Oxford)* 2015; 54:564–565. 25504961 10.1093/rheumatology/keu474

[23] Schroder K Tschopp J The inflammasomes. *Cell* 2010; 140:821–832. 20303873 10.1016/j.cell.2010.01.040

[24] Wilson NJ Boniface K Chan JR Development, cytokine profile and function of human interleukin 17-producing helper T cells. *Nat Immunol* 2007; 8:950–957. 17676044 10.1038/ni1497

[25] Chung Y Chang SH Martinez GJ Critical regulation of early Th17 cell differentiation by interleukin-1 signaling. *Immunity* 2009; 30:576–587. 19362022 10.1016/j.immuni.2009.02.007 PMC2705871

[26] Boraschi D Dinarello CA IL-18 in autoimmunity: review. *Eur Cytokine Netw* 2006; 17:224–252. 17353157

[27] Dinarello CA IL-18: A TH1-inducing, proinflammatory cytokine and new member of the IL-1 family. *J Allergy Clin Immunol* 1999; 103:11–24. 9893178 10.1016/s0091-6749(99)70518-x

[28] Haznedaroglu S Ozturk MA Sancak B Serum interleukin 17 and interleukin 18 levels in familial Mediterranean fever. *Clin Exp Rheumatol* 2005; 23 (4 Suppl 38):S77–S80. 16273770

[29] Romagnani P Maggi L Mazzinghi B CXCR3-mediated opposite effects of CXCL10 and CXCL4 on TH1 or TH2 cytokine production. *J Allergy Clin Immunol* 2005; 116:1372–1379. 16337473 10.1016/j.jaci.2005.09.035

[30] Orgun NN Mathis MA Wilson CB Deviation from a strong Th1-dominated to a modest Th17-dominated CD4 T cell response in the absence of IL-12p40 and type I IFNs sustains protective CD8 T cells. *J Immunol* 2008; 180:4109–4115. 18322221 10.4049/jimmunol.180.6.4109 PMC2677099

[31] Kitching AR Turner AL Wilson GR IL-12p40 and IL-18 in crescentic glomerulonephritis: IL-12p40 is the key Th1-defining cytokine chain, whereas IL-18 promotes local inflammation and leukocyte recruitment. *J Am Soc Nephrol* 2005; 16:2023–2033. 15888563 10.1681/ASN.2004121075

[32] Yoshida A Kohka Takahashi H Iwagaki H Essential role of ICAM-1/LFA-1 interaction in synergistic effect of IL-18 and IL-12 on IFN-gamma production in human PBMC. *Naunyn Schmiedebergs Arch Pharmacol* 2002; 365:181–186. 11882913 10.1007/s00210-001-0518-6

[33] Burne MJ Elghandour A Haq M IL-1 and TNF independent pathways mediate ICAM-1/VCAM-1 up-regulation in ischemia reperfusion injury. *J Leukoc Biol* 2001; 70:192–198. 11493610

[34] Sasaki M Namioka Y Ito T Role of ICAM-1 in the aggregation and adhesion of human alveolar macrophages in response to TNF-alpha and INF-gamma. *Mediators Inflamm* 2001; 10:309–313. 11817671 10.1080/09629350120102325 PMC1781738

[35] Direskeneli H Ozdogan H Korkmaz C Serum soluble intercellular adhesion molecule 1 and interleukin 8 levels in familial Mediterranean fever. *J Rheumatol* 1999; 26:1983–1986. 10493680

[36] Chen DY Lan JL Lin FJ Association of intercellular adhesion molecule-1 with clinical manifestations and interleukin-18 in patients with active, untreated adult-onset Still's disease. *Arthritis Rheum* 2005; 53:320–327. 15934126 10.1002/art.21164

[37] Stojanov S Lapidus S Chitkara P Periodic fever, aphthous stomatitis, pharyngitis, and adenitis (PFAPA) is a disorder of innate immunity and Th1 activation responsive to IL-1 blockade. *Proc Natl Acad Sci U S A* 2011; 108:7148–7153. 21478439 10.1073/pnas.1103681108 PMC3084055

[38] Koga T Umeda M Migita K A Japanese case of familial Mediterranean fever presenting diffuse bone marrow uptake of FDG-PET and high levels of neutrophil membrane CD64 expression. *Rheumatology (Oxford)* 2011; 50:1171–1173. 21296849 10.1093/rheumatology/ker012

[39] Migita K Agematsu K Yamazaki K Expression of CD64 on polymorphonuclear neutrophils in patients with familial Mediterranean fever. *Clin Exp Immunol* 2011; 164:365–372. 21438869 10.1111/j.1365-2249.2011.04380.x PMC3087932

[40] Kawasumi H Gono T Kawaguchi Y IL-6, IL-8, and IL-10 are associated with hyperferritinemia in rapidly progressive interstitial lung disease with polymyositis/dermatomyositis. *Biomed Res Int* 2014; 2014:815245. 24800252 10.1155/2014/815245 PMC3988788

[41] Hamzaoui K Hamzaoui A Guemira F Cytokine profile in Behcet's disease patients. Relationship with disease activity. *Scand J Rheumatol* 2002; 31:205–210. 12369651 10.1080/030097402320318387

[42] Nagaki M Iwai H Naiki T High levels of serum interleukin-10 and tumor necrosis factor-alpha are associated with fatality in fulminant hepatitis. *J Infect Dis* 2000; 182:1103–1108. 10979906 10.1086/315826

[43] Teachey DT Rheingold SR Maude SL Cytokine release syndrome after blinatumomab treatment related to abnormal macrophage activation and ameliorated with cytokine-directed therapy. *Blood* 2013; 121:5154–5157. 23678006 10.1182/blood-2013-02-485623 PMC4123427

[44] Trinchieri G Pflanz S Kastelein RA The IL-12 family of heterodimeric cytokines: new players in the regulation of T cell responses. *Immunity* 2003; 19:641–644. 14614851 10.1016/s1074-7613(03)00296-6

[45] Erken E Ozer HT Gunesacar R Plasma interleukin-10 and interleukin-12 levels in patients with familial Mediterranean fever. *Rheumatol Int* 2006; 26:862–864. 16397779 10.1007/s00296-005-0099-7

[46] Akgul O Kilic E Kilic G Efficacy and safety of biologic treatments in Familial Mediterranean Fever. *Am J Med Sci* 2013; 346:137–141. 23276893 10.1097/MAJ.0b013e318277083b

[47] Yamazaki T Shigemura T Kobayashi N IL-18 serum concentration is markedly elevated in typical familial Mediterranean fever with M694I mutation and can distinguish it from atypical type. *Mod Rheumatol* 2014; 1–3. 25528861 10.3109/14397595.2014.988861

[48] Leung YY Yao Hui LL Kraus VB Colchicine-update on mechanisms of action and therapeutic uses. *Semin Arthritis Rheum* 2015; 45:341–350. 26228647 10.1016/j.semarthrit.2015.06.013 PMC4656054

