# Supplementary figures and images for: Multiple Serum Cytokine Profiling to Identify Combinational Diagnostic Biomarkers in Attacks of Familial Mediterranean Fever
Source: Medicine (Baltimore). 2016 Apr 22;95(16):e3449. doi: 10.1097/MD.0000000000003449 (PMC4845848; doi:10.1097/MD.0000000000003449)

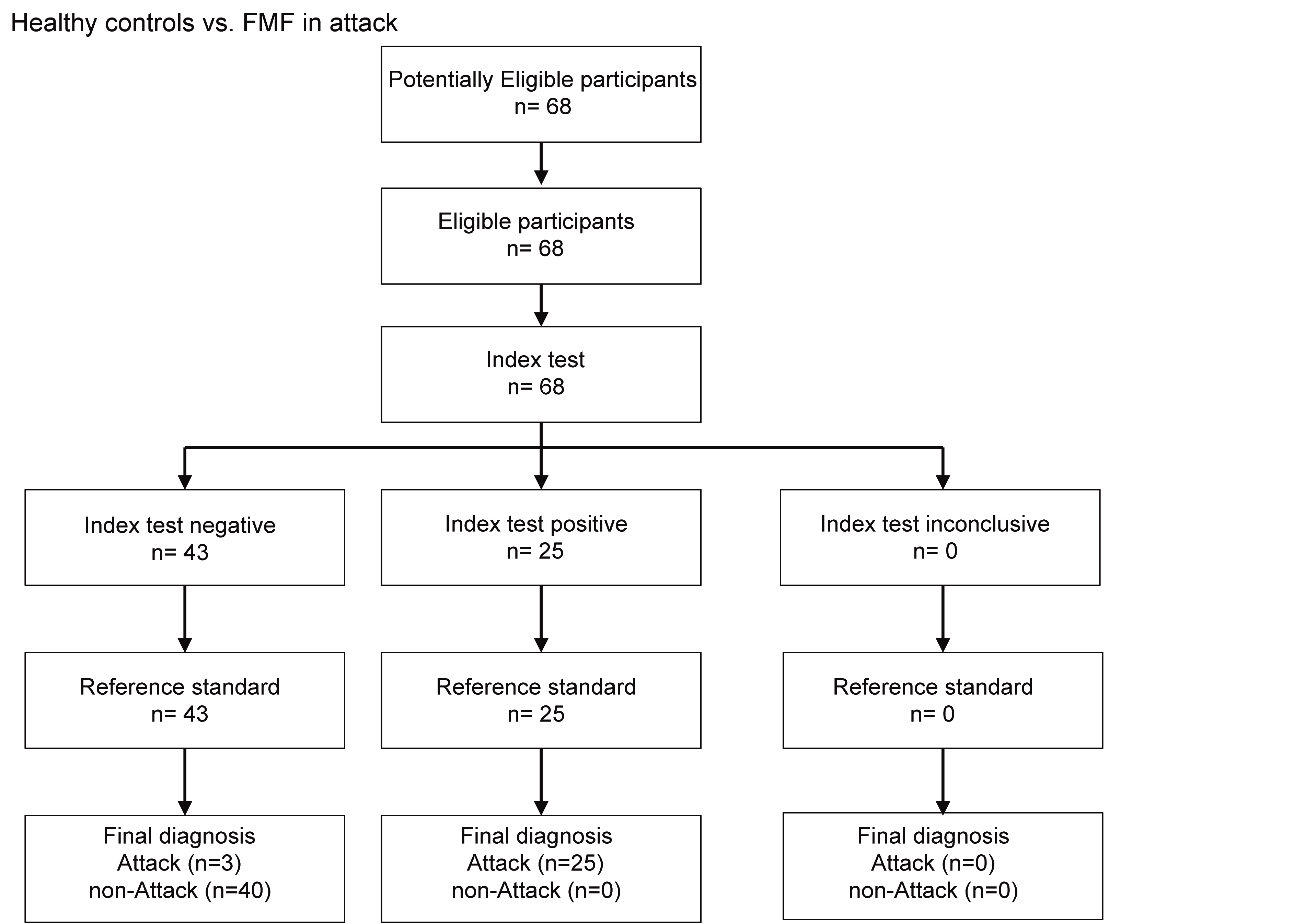


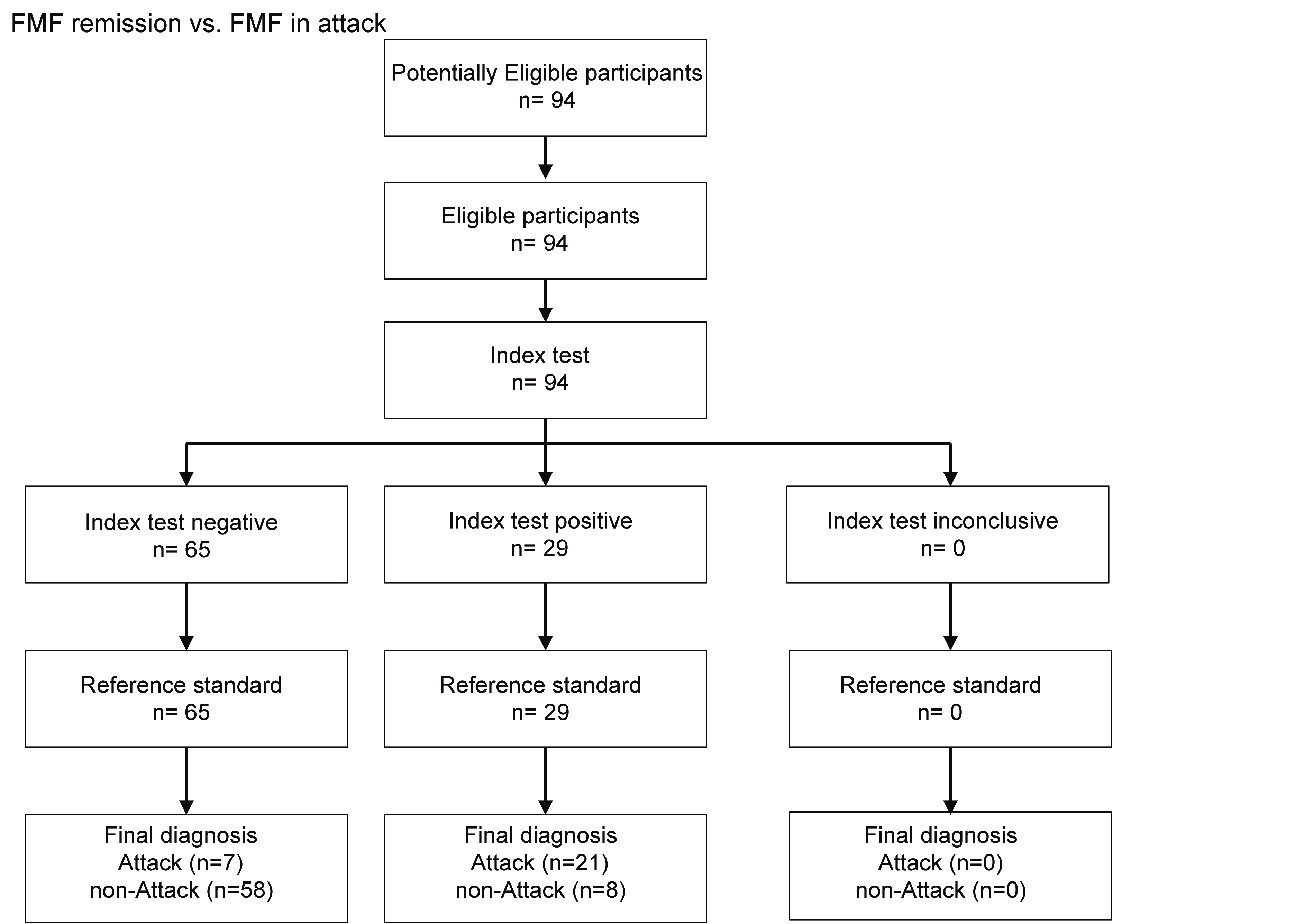

Supplement: Supplemental Digital Content [file medi-95-e3449-s001.doc]
